# A Novel Multi-Digital Camera System Based on Tilt-Shift Photography Technology

**DOI:** 10.3390/s150407823

**Published:** 2015-03-31

**Authors:** Tao Sun, Jun-yong Fang, Dong Zhao, Xue Liu, Qing-xi Tong

**Affiliations:** 1 Key Laboratory of Technology in Geo-spatial Information Processing and Application System, Institute of Electronics, Chinese Academy of Sciences, Beijing 100190, China; 2 National Engineering Research Center for Geo-informatics, Institute of Remote Sensing and Digital Earth, Chinese Academy of Sciences, Beijing 100101, China; E-Mails: zhaodong@radi.ac.cn (D.Z.); lxlaf@163.com (X.L.); 3 State Key Laboratory of Remote Sensing Science, Institute of Remote Sensing and Digital Earth Chinese Academy of Sciences, Chinese Academy of Sciences, Beijing 100101, China; E-Mail: tqxi@263.net

**Keywords:** remote sensing and sensors, geometric calibration, tilt-shift photography, multi-digital camera system

## Abstract

Multi-digital camera systems (MDCS) are constantly being improved to meet the increasing requirement of high-resolution spatial data. This study identifies the insufficiencies of traditional MDCSs and proposes a new category MDCS based on tilt-shift photography to improve ability of the MDCS to acquire high-accuracy spatial data. A prototype system, including two or four tilt-shift cameras (TSC, camera model: Nikon D90), is developed to validate the feasibility and correctness of proposed MDCS. Similar to the cameras of traditional MDCSs, calibration is also essential for TSC of new MDCS. The study constructs indoor control fields and proposes appropriate calibration methods for TSC, including digital distortion model (DDM) approach and two-step calibrated strategy. The characteristics of TSC are analyzed in detail via a calibration experiment; for example, the edge distortion of TSC. Finally, the ability of the new MDCS to acquire high-accuracy spatial data is verified through flight experiments. The results of flight experiments illustrate that geo-position accuracy of prototype system achieves 0.3 m at a flight height of 800 m, and spatial resolution of 0.15 m. In addition, results of the comparison between the traditional (MADC II) and proposed MDCS demonstrate that the latter (0.3 m) provides spatial data with higher accuracy than the former (only 0.6 m) under the same conditions. We also take the attitude that using higher accuracy TSC in the new MDCS should further improve the accuracy of the photogrammetry senior product.

## 1. Introduction

Multi-digital camera systems (MDCSs) are constantly being improved to meet the increasing requirement of high-resolution spatial data. The MDCSs constitute a class of optical sensors and is composed of a variety of devices, including multi-cameras, global navigation satellite system (GNSS), inertial measurement unit (IMU) and others (e.g., MADC II [1], SWDC [2], DMC [3] and UltraCam [4]). Part of the current interest in developing MDCSs stems from the limitations in the size of current Charge-coupled Device (CCD) and Complementary Metal-Oxide-Semiconductor (CMOS) area arrays for digital cameras—hence the use of multiple cameras and other auxiliary devices by a number of system suppliers to increase the ground area that can be covered from a single exposure station. Besides the increase in the coverage area of rectangular or square format digital frame photos, there is a long-standing requirement on the part of earth observation, survey, military and many fields to obtain the widest possible cross-track angular coverage using MDCSs. Apart from the multi-photo and multi-camera aspects of photography, the MDCSs are also of increasing importance for both surveillance and for visualization purposes—with the acquisition of multiple digital images from both manned and unmanned platforms. The advantages of MDCSs can facilitate the rapid acquisition of high-resolution spatial data. As such, MDCSs is widely used in photogrammetry engineering and research (e.g., [5,6,7,8,9,10]).

**Figure 1:**
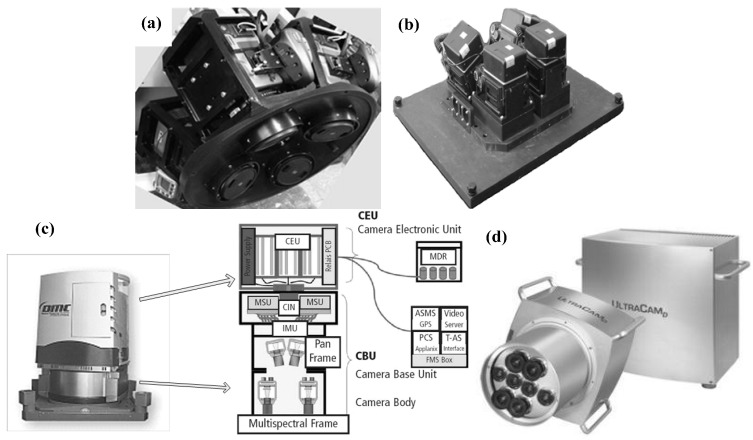
Exiting MDCSs: (**a**) SWDC-4; (**b**) MADC II; (**c**) DMC; and (**d**) UltraCam.

Fifteen years ago the first commercial MDCS (DMC, Intergraph/ZI-Imaging, Aalen, Germany) was released for sale. Since then the development of MDCSs have become an open issue. The UltraCam (Vexcel Corp., Boulder, CO, USA) and DiMAC (DiMAC Systems) have to be mentioned as latecomers in the field of MDCS providers. They have been commercially available for almost ten years. In the beginning of 2005, several tests with multiple camera modules as well as different lenses were performed and corresponding reports were published. The basic concept of MDCS was gradually built. In China, the study of MDCSs, SWDC and MADC specifically, were begun in 2008 and 2007, respectively. Although many types of MDCS exist in the field of photogrammetry, the composition of existing MDCSs can be divided into two categories: MDCS based on tilt photography and MDCS based on delay exposure. In tilt photography technology-based MDCS, some or all of its cameras are arranged in a tilted position, as shown in Figure 1a (SWDC), Figure 1b (MADC II) and Figure 1c (DMC). The accuracy of these MDCSs, especially those whose cameras are in a tilted position, suffers from the problem of projection differences (PDs). High-accuracy digital elevation model (DEM) data can be used to address this problem (*i.e*., to correct PDs), but the quality of the additional DEM data still determines the accuracy of the correction. Another problem that commonly occurs in such cameras is the difficulty of achieving the unified spatial resolution of an image captured from each camera. More details on PD and its impact on the geometric accuracy of MDCSs can be found in [11,12]; a processing method that addresses some of the common problems in DMC and MADCII is introduced in [13,14,15,16]. In delay exposure technology-based MDCS, as represented by UltraCam [17] (Figure 1d), the exposure of each camera is short-time asynchronous. The accuracy of these MDCSs depends not only on the punctuality of the shutter’s control system but also on the stability of the platform in a given period of time for a group of images. Long-periodic attitude and position deviations can be corrected by high-accuracy GNSS and IMU devices, but short-periodic deviations caused by the instability of the platform (e.g., platform jitter) cause great difficulties in data processing. Some processing methods that address some of these problems (e.g., platform instability) in UltraCam can be found in [18,19].

Kass [20] conducted an experiment to test the geometric accuracy of DMC, UltraCam and ADS40 under the same conditions. The experiment showed that the accuracy ranking of the cameras from high to low is as follows: ADS40, DMC and UltraCam. To further improve the geometric accuracy and simplify the image process of MDCS, the present study proposes a third category of MDCS: the tilt-shift photography technology-based MDCS. The advantages of the new MDCS are the following: it eliminates the need to arrange the cameras in a tilted position, and it makes all cameras synchronous in capturing images (synchronous exposure). The image process of the new MDCS also eliminates the need to correct PDs and unify the spatial resolution of the images captured from each camera; the stability of the platform cannot be considered because of synchronous exposure.

However, geometric calibrations are necessary in both the traditional and our proposed MDCS. Moreover, the distortion of the edges of images captured by cameras with tilt-shift photography (*i.e*., tilt-shift camera (TSC)) is larger than that of traditional cameras. Therefore, further research on the appropriate calibration technique for TSC is crucial. The contributions of the present work are the following: (1) a novel MDCS; (2) a geometric calibration for TSC camera and experiments; and (3) image processing and accuracy assessment using flight experiments for the new MDCS.

The rest of this paper is organized as follows: Section 2 introduces the principle of tilt-shift photography and the novel MDCS and prototype system developed in this study. Section 3 and Section 4 discuss the indoor control field-based calibration methods for the TSC and experiments, respectively. Section 5 discusses the flight experiments conducted on the new MDCS and MADCII. Section 6 ends with the conclusions of the study.

## 2. Novel MDCS and Its Prototype System

### 2.1. Principle of Tilt-Shift Photography Technology

Tilt-shift photography technology is often used in cameras lenses, which encompasses two different types of movements: the rotation of the lens plane relative to the image plane, called tilt, and the movement of the lens plane parallel to the image plane, called shift. Tilt-shift lenses are designed to emulate the camera movements found on larger monorail and technical field cameras. These cameras, with their fine mechanical controls, have the ability to move this slice of space to the left and right, up and down and most importantly tilt and swing it away from the perpendicular.

**Figure 2:**
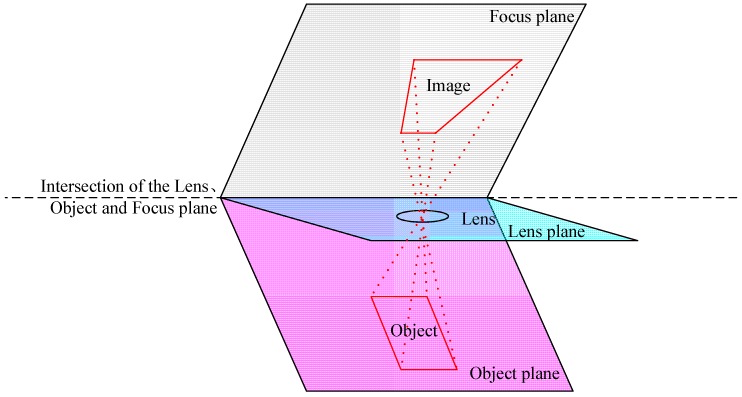
A simplified diagram of the Scheimpflug rule.

Tilt is used to control the orientation of the focus plane (FP) and therefore the part of the image that appears sharp; it follows the Scheimpflug principle [21,22]. As shown in Figure 2, this is a simplifiedillustration of the Scheimpflug rule. Theodor Scheimpflug stated that: “when the extended lines from the lens plane, the object plane and the film plane intersect at the same point, the entire subject plane is in focus”. 

**Figure 3:**
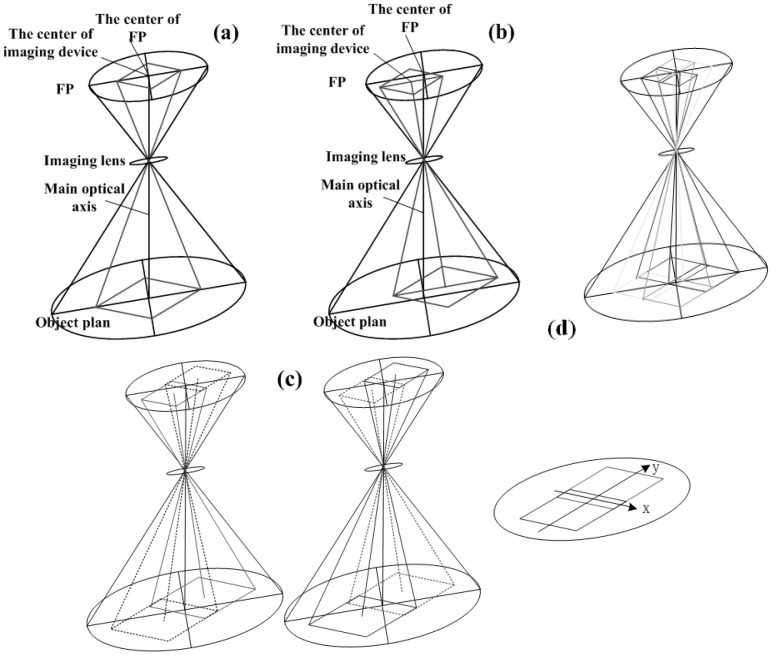
Intuitive representation of the imaging mode and novel MDCS: (**a**) traditional imaging mode; (**b**) tilt-shift imaging mode; and (**c**,**d**) novel MDCS, which include two or four chip CCD arrays.

Shift is used to adjust the position of the subject in the image area without moving the camera back; this function is often helpful in avoiding the convergence of parallel lines, as in the case of photographing tall buildings. In this paper, we mainly use Shift function of tilt-shift lens to composite the novel MDCS. Figure 3b below illustrates the concept of Shift function. More details on tilt-shift photography technology are found in [23,24,25,26], and a description of tilt-shift lens can be found in [27,28,29].

### 2.2. Novel MDCS

The novel MDCS fully uses the tilt-shift imaging mode (TSIM) derived from tilt-shift technology. Figure 2 shows an intuitive representation of the camera composition of this MDCS. In a traditional camera, the main optical axis (MOA) is perpendicular to the FP, and the intersecting point is located at the center of the CCD arrays, as shown in Figure 3a. With respect to TSIM, lenses that use tilt-shift technology are utilized to shift the location of the intersecting point of MOA and FP at the edge of the CCD arrays, as shown in Figure 3b. According to the characteristics of TSIM, multi-chip CCD arrays can be located in the FP, as shown in Figure 3c,d. By sharing tilt-shift lenses, these CCD arrays can separately capture different regional terrains during camera exposure, thereby achieving the objective of a wide imaging field.

The new MDCS is better than the older MDCSs, because in the former all the cameras are arranged in a vertical position and all of them synchronously capture images. This principle of arrangement implies the following: (1) Compared with the first category of MDCS introduced in Section 1 (e.g., MACD II), the new MDCS can prevent some problems, such as projection difference and different image resolution, caused by tilted photography in the old MDCSs; and (2) compared with the second category of MDCS (e.g., UltraCam), the new MDCS does not need high-accuracy GNSS and IMU devices (which are too expensive) to correct attitude and position deviations caused by the platform instability during the period of short-time asynchronous exposure.

### 2.3. Prototype System of Novel MDCS

**Figure 4:**
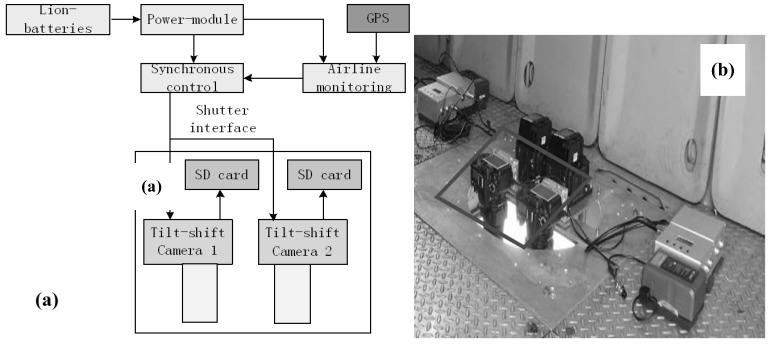

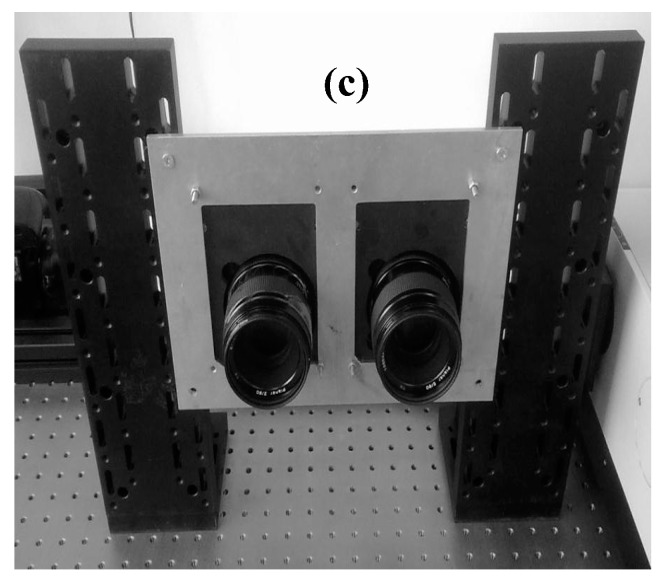
Prototype system of the novel MDCS: (**a**) structure diagram; (**b**) installation in the Yun-5 aircraft for flight experiment; and (**c**) installation in the optical platform for calibration.

The diagram in Figure 4a shows a prototype system of the novel MDCS, including the multiple tilt-shift cameras (camera model: Nikon D90), synchronous control system and GNSS. This prototype was built to verify the feasibility of the novel MDCS; its parameters are listed in the Table 1.

**Table 1:** Parameters of Prototype System of novel MDCS.

	Array Size	Value of Tilt-Shift (mm)	Field Angle (°)	Focal Length (mm)	Pixel Size (μm)	Base-Height Ratio (Course Overlap: 60%)
**Single TSC**	4288 × 2848	0	52° × 36°	24	5.5	0.4
**Two TSCs (Extension by short edge)**	4200 × 5410	±7	52° × 63°	24	5.5	0.5
**Two TSCs (Extension by long edge)**	8290 × 2800	±11	87° × 36°	24	5.5	0.76
**Four TSCs**	7910 × 4960	±5.7, ±10	84° × 60°	24	5.5	0.73

The overlapping pixel number of the prototype system using two TSCs is about 300 pixels, while that of the prototype system using four TSCs is 700 pixels. The synchronous control system permits the synchronous exposure of all the cameras, and the shortest time interval is 2 s. The TSC has a storage capacity of about 2000 images; it can therefore continuously work for 3 h at a flight speed of 180 km/h, flight height of 1000 m and course overlap of 60%.

The data in Table 1 shows that the composition of TSC significantly increases the field angle and base-height ratio of the device, while keeping all the cameras in a vertical position. The prototype system using two TSCs with extension by short edge is used as the experimental equipment in the following section. As Figure 4b,c show, the prototype system is installed in a Yun-5 aircraft for the flight experiment and in the optical platform of the indoor control fields for calibration.

## 3. Calibration Methods for TSC Based on Indoor Control Field

The targets of the indoor geometric calibration for TSC, including the interior orientation elements (IOEs) and distortion, are similar to those for the traditional camera. The calibration strategy is undertaken in two steps to separate the calibration for the IOEs and that for the distortion, thereby avoiding additional operations and specific conditional requirement (e.g., spatial depth, tilted images and suitable ray intersections) for mitigating the problems of over-parameterization and correlations between parameters in the one step calibration processing for all elements. An improved digital distortion model (DDM) method is used in the calibration for the distortion characteristics of TSC.

### 3.1. Indoor Control Fields

**Figure 5:**
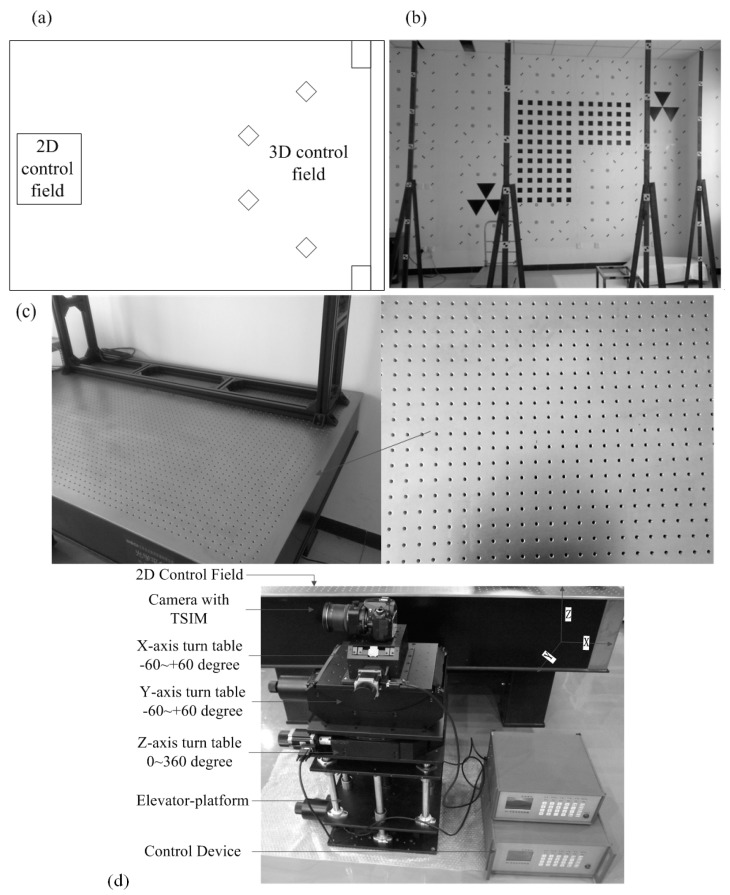
(**a**) Indoor calibration field; (**b**) 3DCF; (**c**) 2DCF and OP; and (**d**) tree-axis turntables and tilt-shift camera.

The indoor control fields consist of the 3D and 2D control fields (2DCF and 3DCF) and the tree-axis turntables, as shown in Figure 5a. A total of 400 high-accuracy control points are located in the various planes in the 3DCF, while a kind of rigid structure located at the top of the optical platform (OP) constitutes the 2DCF, as shown in Figure 5b,c. Using the forward intersection and indirect elevation methods [30], all the points in the 3DCF and 2DCF are surveyed by 2'-transit (*i.e*., the accuracy is 2'). The precision of the control points can be assessed according to the precision of the transit, survey distance and other aspects [30]. The precision of the control points is shown in Table 2.

**Table 2:** Precision of the 2D and 3D control points (mm).

	Plane Precision	Elevation Precision
2DCF	1.1	1.8
3DCF	2.3	3.4

The tree-axis turntables are used in the calibration process for DDM; the combination of TSC and turntables is shown in Figure 5d. The precision of each turntable is listed in Table 3. The OP and equipment of the tree-axis turntables are provided by Beijing Optical Century Instrument Co., Ltd. (Beijing, China), and the specification and information are available from the public domain website [31]. 

**Table 3:** Precision of each turntable (°).

Direction	Equipment Model	Repeat Precision	Resolution
X-axis	MGC103	0.002	0.0001
Y-axis	MGC105	0.001	0.00005
Z-axis	MRS105	0.003	0.000039

### 3.2. DDM

In the tilt-shift camera, the magnitude of the distortion away from the tilt-shift direction is theoretically bigger than that close to the tilt-shift direction. We therefore assume that the distortion of TSC is not similar to that of the traditional camera whose model can be built by some distortion coefficients (e.g., k1, k2, p1, p2, *etc.*). More details about calibration functional models based on distortion coefficients can be found in Section 7 of [30]. We also regard the DDM as perhaps the best distortion model for TSC. DDM [32] is a kind of 3D model that is similar to DEM. The plane coordinate grid of DDM represents the ranks and arrays of CCD, and the elevation represents the corresponding distortion. When the DDM of the camera is acquired, the distortion correction becomes a simple matter of processing which directly corrects the distortion pixel by pixel. The important part of processing is the acquisition of DDM. Feng [32] proposes an acquisition method for DDM premised on the artificial assumption that regards the distortions of image corners as zero. This assumption is inappropriate because the distortions of image corners are theoretically the biggest [33].

**Figure 6:**
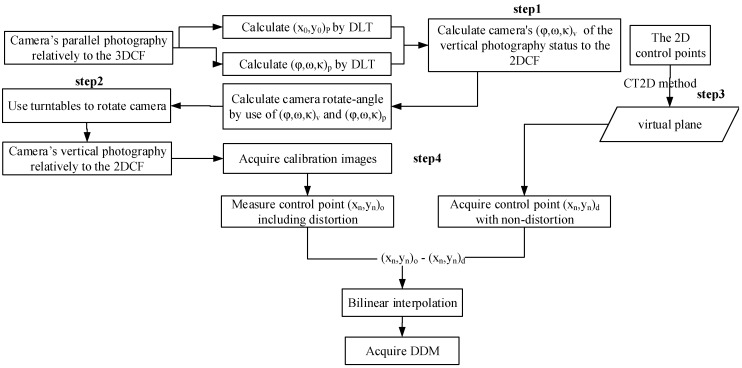
IDAM process.

To avoid this artificial assumption, our method uses the tree-axis turntables and 3DCF—that is, the improved DDM acquisition method (IDAM)—in calculating camera’s DDM; the processing is described in Figure 6.

First, the 3D control field and DLT algorithm are used to calculate calibrated camera’s relative orientation in the coordinate system. Second, the tree-axis turntables are utilized to ensure that the cameras achieve their designated status, that is, their main optical axis is perpendicular to the plan of 2DCF. Thirdly, algorithms that transform the 2D control points to virtual plane (CT2D) [34,35] are used to eliminate the feasible existing projection distortion. Finally, the DDM can be directly acquired based on the relationship of the CP position with non-distortion and that of the CP position with the distortion in the image.

### 3.3. Calibration Methods for IOEs

IOEs include the principal point (x_0_, y_0_) and focal length *f*. The algorithms for calibrating the IOEs of the camera have been proposed in many studies. To calculate the IOEs, we adopt one of these algorithms, namely the direct linear transformation (DLT) algorithm. DLT is a common calibration method via directly building relationship between imagery coordinates and object coordinates; therefore, the initial value of IOEs is not necessary during the calculation. Given the difficulty in acquiring appropriately initial value of IOEs in TSC, DLT is one of appropriate methods for the interior parameter recovery of TSC.

As described in Section 7 of [30], DLT’s normal equation and error equation are shown in Equation (1):

(1) $$\left\{ \begin{array}{l} V=ML-W\\ L={\left(M^{T}M\right)}^{-1}M^{T}L \end{array}\right.$$

In which:

$$V={\left[v_{x},v_{y}\right]}^{T}$$

$$M=-\left[\begin{array}{l} \frac{X}{A}\;\frac{Y}{A}\;\frac{Z}{A}\;\frac{1}{A}0\;0\;0\;0\;\frac{xX}{A}\;\frac{xY}{A}\;\frac{xZ}{A}\\ 0\;0\;0\;0\;\frac{X}{A}\;\frac{Y}{A}\;\frac{Z}{A}\;\frac{1}{A}\;\frac{yX}{A}\;\frac{yY}{A}\;\frac{yZ}{A} \end{array}\right]\;$$

$$L={\left(l_{1},l_{2},l_{3},...,l_{11}\right)}^{T}$$

$$W={\left[-\frac{x}{A},-\frac{y}{A}\right]}^{T}$$

The L-coefficients can be obtained based on least-squares principle via setting up enough error equations by use of multiple control points. Consequently, IOEs are the linear composition of those L coefficients, the details of which can be found in [30,36].

## 4. Calibration Experiments for TSC and Analysis of the Results

**Figure 7:**
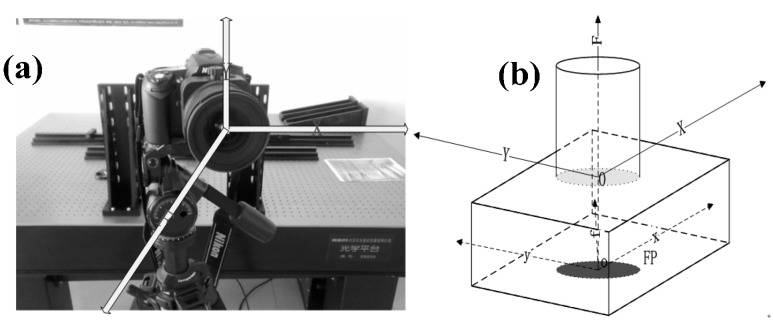
(**a**) TSC of the prototype system and (**b**) the defined coordinates.

The TSCs of the prototype system (Nikon D90 and tilt-shift lens) have two kinds of shift-directions: horizontal (left-right) and vertical (up-down) (Figure 7a). The objective of the calibration experiment is to calibrate the DDM and IOEs of TSC under different tilt-shift conditions and to validate the correctness and feasibility of our calibration methods. To facilitate the follow-up description, this study defines the coordinates (*XOY*−*F*) of TSC, as shown in Figure 7b. In this figure, the horizontal shift-direction is the *X*-axis and the right-direction is positive; the vertical shift-direction is the Y -axis and up-direction is positive; the *F*-axis is the direction of the main optical axis; the coordinate’s origin *O* is the intersection of the *F*-axis and plane-*XOY* in the case of zero tilt-shift condition. The coordinates (*xoy*−*f*) constitute the image plane coordinate system.

The three categories of the calibration experiment for the TSC are as follows.

(1) Tilt-shift and the nine groups of experiments: one group of experiments is conducted under the zero tilt-shift condition, four groups under the condition with different tilt-shift values along the Y direction, and four groups along the X direction.
(2) Restarting the cameras, including two groups of experiment: one group of experiments is conducted under the zero tilt-shift condition, and another group under the largest tilt-shift magnitude along the Y direction.
(3) Lens reshipment, including two groups of experiment: one group of experiments is conducted under the zero tilt-shift condition, and another under the largest tilt-shift magnitude along the Y direction.

With the help of the calibration method discussed in Section 3, the DDM of the TSC is calculated, after which the DLT is used to calculate the IOEs of the TSC. The checkpoint method is used to validate the accuracy of the calibration. The number of checkpoints and RMSE are listed in Table 4, Table 5 and Table 6.

### 4.1. Results of the Tilt-Shift Experiment

The results in Table 4 (the IOEs of TSC in the tilt-shift experiment) show that the focal length of TSC is unstable under the condition of different tilt-shift value and that the largest change in the focal length is 0.106 mm (with a relative error of 1/235). We suggest building a lookup table of focal length in the indoor control field to ascertain the value of the focal length under the condition of different tilt-shift value.

**Table 4:** IOEs of the camera in the tilt-shift experiment (mm).

Tilt-Shift	∆*m_X_*	∆*m_Y_*	*x*_0_	*y*_0_	*f*	∆*m_x_* *	∆*m_y_* *	∆*f* *	No. of Checkpoints	RMSE (Pixels)
Zero	0	0	11.99979	8.86909	25.0698	0	0	0	43	0.273
Along the Y direction	0	5.2	12.02504	14.06404	25.05845	0.02525	5.194953	–0.011352	36	0.364
	0	7.2	12.03491	16.06058	25.05385	0.03512	7.191487	–0.015948	42	0.168
	0	–5.2	11.95968	3.649679	25.04293	–0.04011	–5.21941	–0.026866	46	0.384
	0	–10.8	11.93393	–1.9371	24.97507	–0.06586	–10.8062	–0.094724	51	0.453
Along the X direction	4.8	0	16.79214	8.82381	25.11648	4.79235	–0.02511	0.046686	23	0.268
	10.8	0	22.80507	8.84398	25.13975	10.80528	–0.04528	0.069951	31	0.478
	–6	0	5.994606	8.89152	25.06754	–6.00519	0.02243	–0.002258	16	0.426
	–12	0	–0.02098	8.90431	24.96335	–12.0208	0.03522	–0.10645	34	0.359

* ∆*m_x_* is equal to *x*_0_ of the tilt-shift situation minus *x*_0_ of the zero tilt-shift situation; ∆*m_y_* is equal to *y*_0_ of the tilt-shift situation minus *y*_0_ of the zero tilt-shift situation; **∆***f* is equal to *f* of the tilt-shift situation minus *f* of the zero tilt-shift situation.

In Table 4, ∆*m_X_*, ∆*m_Y_* represent the theoretical value of the tilt-shift as directly read in the dial of the Lens; (*x*_0_, *y*_0_, *f*) represent the calibrated IOEs of TSC; ∆*m_x_*, ∆*m_y_* represent the calibrated value of the tilt-shift; ∆*f* represents the change of the focal length between the adjacent values of the tilt-shift.

**Figure 8:**
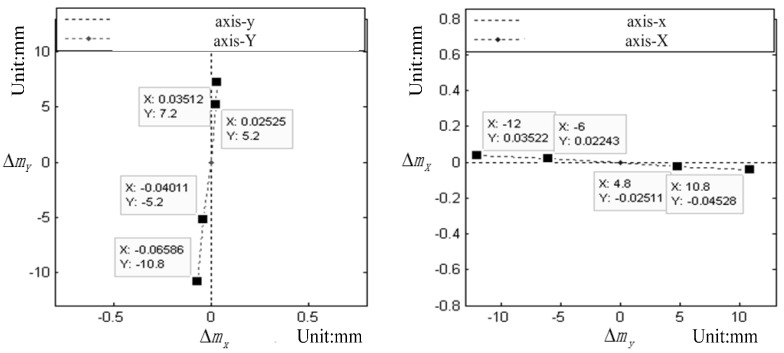
(**a**) Existing angle between the Y-axis and y-axis and (**b**) existing angle between the X-axis and x-axis.

**Figure 9:**
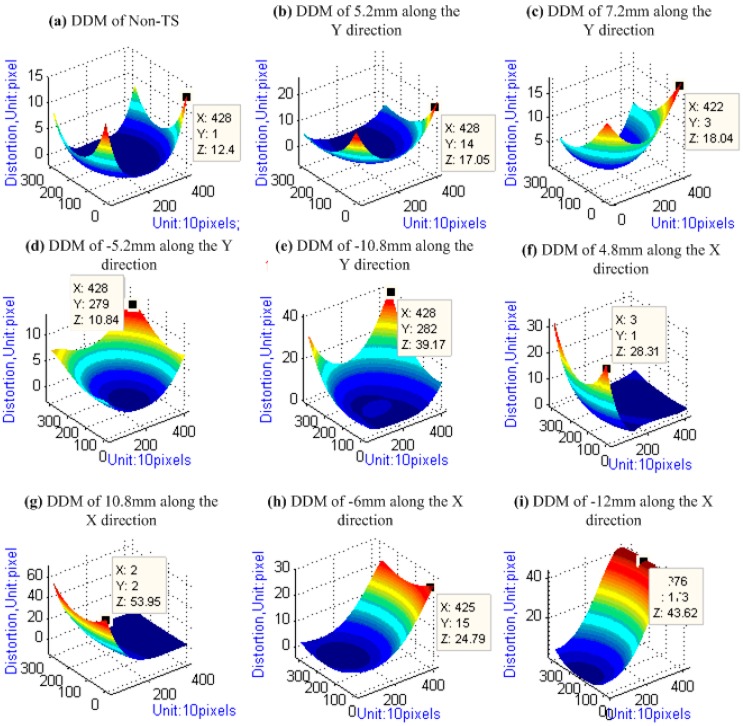
DDM of TSC: (**a**) DDM under the zero tilt-shift condition; (**b**–**e**) DDM under the tilt-shift along the Y direction; and (**f**–**i**) DDM under the tilt-shift along the X direction. Pixel size: 5.5 μm.

As further described by the calibrated data in Table 4, Figure 8 illustrates that the actual mechanical tilt-shift direction (X-axis, Y-axis) does not coincide with the image plane coordinate system (x-axis, y-axis). This characteristic must be considered in the TSC calibration.

The results of the DDM of the TSC in the tilt-shift experiment are shown in Figure 9, where the x-axis and y-axis (unit: 10 pixels) represent the ranks and arrays of CCD, respectively, and the z-axis represents the total distortion (unit: 1 pixel). The results show that the magnitude of the distortion away from the tilt-shift direction is larger than that close to the tilt-shift direction. The magnitude of the edge distortion increases with increasing tilt-shift value. Thus, for distortion expression and correction, DDM is better than the distortion coefficients.

### 4.2. Results of the Camera Restarting Experiment

**Figure 10:**
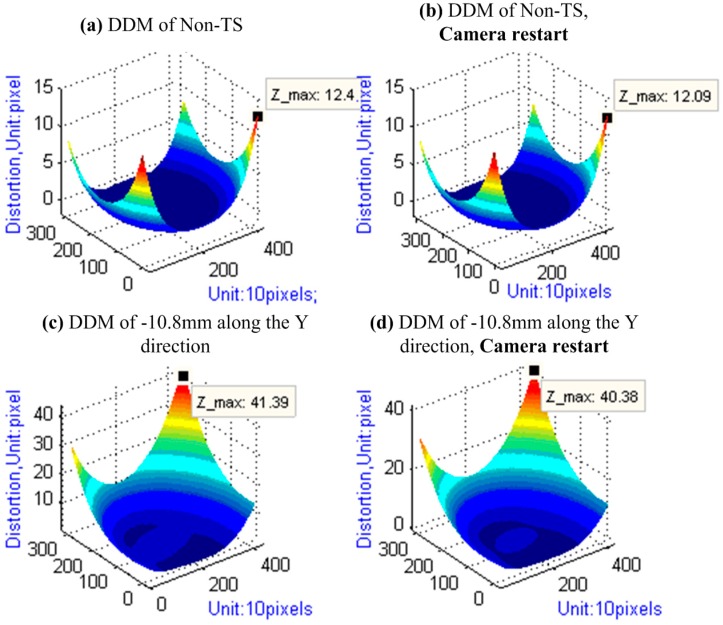
DDM of Camera Restart experiment.

The results in Table 5 and Figure 10 show that, when restarting the camera, the changes in the IOEs under the large tilt-shift value are larger than those under the zero tilt-shift value. However, the magnitude of the deviation is very subtle so that it can be neglected.

**Table 5:** IOEs of the camera in the restarting experiment (mm).

Tilt Shift (mm)	Camera Restart	*x*_0_ (mm)	*y*_0_ (mm)	*f* (mm)	No. of Checkpoints	RMSE (Pixel)	Note
0		11.99979	8.86909	25.0698	46	0.583	1(pixel) = 0.0055 (mm)
	Restart	12.00549	8.86479	25.0686	29	0.429	
10.8		11.93394	−2.0271	24.9751	37	0.366	
	Restart	11.94504	−2.0385	24.9893	19	0.453	

### 4.3. Results of Lens Reshipment Experiment

**Figure 11:**
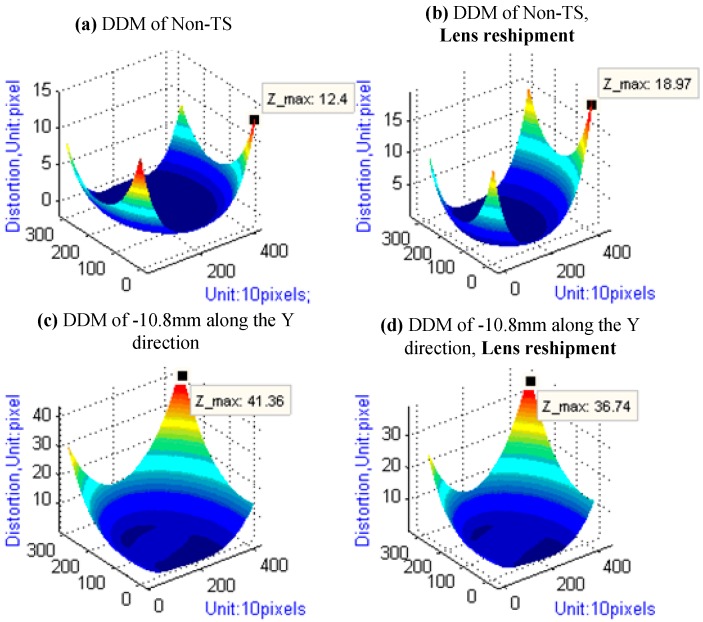
DDM of the lens reshipment experiment.

As shown in Table 6 and Figure 11, the results illustrate that Lens Reshipment brings very great impact on IOEs and Distortion of camera either under the tilt-shift condition or not. The maximum deviation of principal point, focal length, and maximum distortion are about 11, 11, and 4.5 pixels under the tilt-shift condition, respectively. Thus, calibration needs to be redone if tilt-shift lens reship.

**Table 6:** Camera’s inner orientation elements in the lens reshipment experiment, unit: mm.

Tilt Shift (mm)	Lens Reshipment	*x*_0_ (mm)	*y*_0_ (mm)	*f* (mm)	No. of Checkpoints	RMSE (Pixels)
0		11.99979	8.86909	25.0698	34	0.368
	reshipment	12.02049	8.82265	25.0516	43	0.345
10.8		11.93394	–2.0271	24.9751	22	0.442
	reshipment	11.99504	–2.0845	24.9293	17	0.328

## 5. Flight Experiment

To experimentally verify the accuracy of the novel MDCS, some flight experiments were performed by installing the prototype system using two TSCs with extension by short edge in the Yun-5 aircraft. The experimental area covers Guangxi Wuzhou, China, which is located in the eastern longitude 111°51′14″ to 111°40′00″ and northern latitude 22°58′12″ to 24°10′14″. The flight height, course overlap and Ground Sample Distance (GSD) in the experiment are 800 m, 60% and about 0.15 m, respectively.

The prototype system in the experiment received 150 groups of images. The steps of the imaging process may be outlined as follows. First, the DDM is calibrated through the IDAM method, and each TSC is subjected to distortion correction. Second, equivalent IOEs of a virtual camera composed by all TSC are constructed according to the calibrated IOEs of each TSC. Third, the matching points derived from the pixel-level phase correlation algorithm [37] and the equivalent IOEs are used to generate the stitched image. Fourth, radiometric correction is performed to eliminate the radiometric difference among the images from the different TSCs, using the method proposed in [38]. The stitched image from a group of images can then be regarded as one image captured from this virtual camera, and the equivalent IOEs constitute the parameters of this camera. Finally, mature photography software (Pixel Grid-ATT) is used for the subsequent processing, which includes block adjustment, DEM generation [39] and so on. Information on Pixel Grid-ATT is available in the public website [40]. 

In the subsequent processing, self-calibration method and GNSS data are used in the block adjustment processing. Given the systematic errors of GNSS, location model changes to Equation (2):

(2) $$\left[\begin{array}{l} X_{A}\\ Y_{A}\\ Z_{A} \end{array}\right]=\left[\begin{array}{l} X_{S}\\ Y_{S}\\ Z_{S} \end{array}\right]+R\left[\begin{array}{l} u\\ v\\ w \end{array}\right]+\left[\begin{array}{l} a_{X}\\ a_{Y}\\ a_{Z} \end{array}\right]+(t-t_{0})\left[\begin{array}{l} b_{X}\\ b_{Y}\\ b_{Z} \end{array}\right]$$

where, [*a_X_ a_Y_ a_Z_*]*^T^* is the distance between the photography center and GNSS center; (*t*−*t*_0_) [*b_X_ b_Y_ b_Z_*]*^T^* is the systematic errors of GNSS. Consequently, linearized error equation based on GNSS observations is built, as shown in Equation (3):

(3) $$\left[\begin{array}{l} V_{X_{A}}\\ V_{Y_{A}}\\ V_{Z_{A}} \end{array}\right]=\frac{\partial X_{A},Y_{A},Z_{A}}{\partial \phi ,\omega ,\kappa }\left[\begin{array}{l} \Delta \phi \\ \Delta \omega \\ \Delta \kappa \end{array}\right]+\left[\begin{array}{l} \Delta X_{S}\\ \Delta Y_{S}\\ \Delta Z_{S} \end{array}\right]+R\left[\begin{array}{l} \Delta u\\ \Delta v\\ \Delta w \end{array}\right]+\left[\begin{array}{l} \Delta a_{X}\\ \Delta a_{Y}\\ \Delta a_{Z} \end{array}\right]+(t-t_{0})\left[\begin{array}{l} \Delta b_{X}\\ \Delta b_{Y}\\ \Delta b_{Z} \end{array}\right]-{\left[\begin{array}{l} X_{A}\\ Y_{A}\\ Z_{A} \end{array}\right]}_{Cal}+{\left[\begin{array}{l} X_{A}\\ Y_{A}\\ Z_{A} \end{array}\right]}_{Obs}$$

An error equation based on the GNSS observations, as shown in Equation (4), is added to the self-calibration error equations, thereby performing block adjustment processing. Given the same observed condition, each weight of observations is set equal. According to self-calibration based on the GNSS observations, equivalent IOEs of prototype system are re-evaluated in the bundle adjustment starting from the values computed in the previous-phase. Finally, the DEM and other product can be generated.

(4) $$V_{gps}=\bar{A}t+Rr+Dd-l_{gps}\;Weight:P_{gps}$$

**Figure 12:**
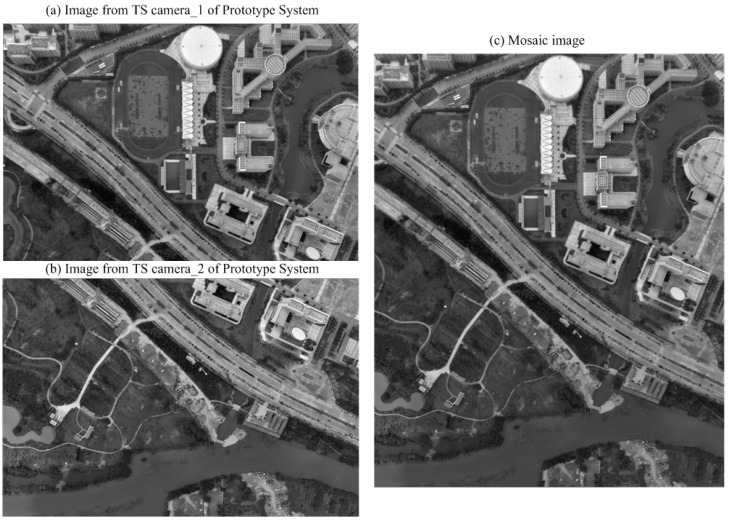
Results of the stitching: (**a**,**b**) image captured from TSCs and (**c**) stitched image.

**Figure 13:**
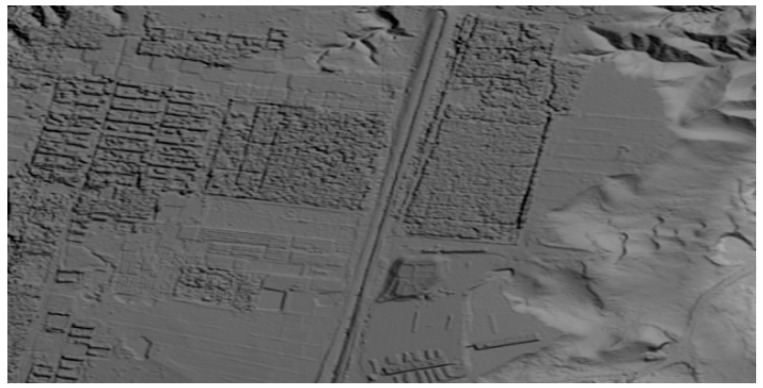
Rendering image of the generated DEM.

Figure 12 and Figure 13 show one of the results of the stitched image and the corresponding regional rendering image of the generated DEM.

**Figure 14:**
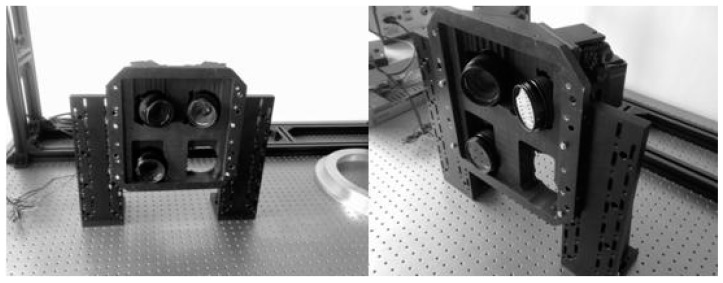
MADCII is set in the optical platform for the calibration of the IOEs and distortion of each camera.

MADC II (*i.e*., an older MDCS) is also installed in the Yun-5 aircraft for comparison. The geometric characteristics of MADCII are listed in Table 7. To ensure a credible assessment for the geometric comparison between MADCII and the prototype system, MADCII is also subjected to geometric calibration and image processing under the same conditions, such as the same indoor control field and Pixel Grid-ATT software. As Figure 14 shows, MADCII is set in the optical platform for the calibration of the IOEs and distortion of each camera. The experimental flight height for MADCII is higher (about 1330 m) than that for the prototype system, thereby ensuring that the spatial resolutions of the image captured from MADCII are approximately equal to those of the prototype system.

**Table 7:** Parameters of TSC and prototype system.

	Array Size	Pixel Size (μm)	Field Angle (°)	Focal Length (mm)	Dip Angle of the Camera in the Tilt Position
Single Camera	4096 × 4096	9	26° × 26°	80	±23°
MADC II	12000 × 4000		72° × 26°		

**Figure 15:**
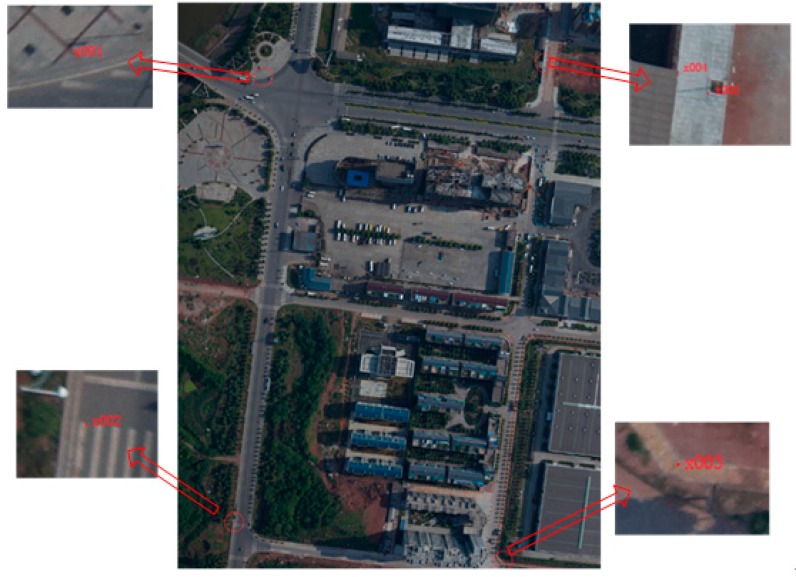
A slice of ground control points.

In the accuracy assessment, we adopt the checkpoint method and prepare 30 checkpoints (CPs) surveyed by the static GNSS (Ashtech ProMark2). The level precision of the GCPs can reaches 6 mm, and the vertical precision can reaches 12 mm by GNSS phase observations of 20 min. The distribution and location of a slice of CPs are shown in Figure 15. The results of the geometric comparison between MADCII and the prototype system are listed in Table 8.

In another experiment, we use the following two sets of distortion parameters to separately perform image processing: those calibrated through our proposed DDM method and the traditional distortion coefficients method acquired from a one-step calibration. In traditional distortion coefficients method, we adopt four coefficients, including radial distortions (k1 and k2) and tangential distortions (p1 and p2), to describe the camera’s distortion. Distortion model based on four coefficients is shown as Equation (5). This experiment is used for the validation of the accuracy and feasibility of the DDM method, and the results are also listed in Table 8.

(5) $$\left\{ \begin{array}{l} \Delta x=(x-x_{0})(k_{1}r^{2}+k_{2}r^{4})+2p_{1}(x-x_{0})(y-y_{0})+p_{2}(r^{2}+2{\left(x-x_{0}\right)}^{2})\\ \Delta y=(y-y_{0})(k_{1}r^{2}+k_{2}r^{4})+2p_{1}(x-x_{0})(y-y_{0})+p_{1}(r^{2}+2{\left(y-y_{0}\right)}^{2})\\ r^{2}={\left(x-x_{0}\right)}^{2}+{\left(y-y_{0}\right)}^{2} \end{array}\right.$$

**Table 8:** Accuracy of the prototype system and MACDII (m).

	Ground Sample Distance	Amount of CPs	Distortion Correction Using DDM		Distortion Correction Using Distortion Coefficients	
			RMS of CPs (Plane Precision) *	RMS of CPs (Height Precision) ^+^	RMS of CPs (Plane Precision) *	RMS of CPs (Height Precision) ^+^
prototype system	about 0.15	30	**0.28**	**0.32**	0.83	1.21
MACD II	about 0.15	30	0.54	0.63	1.06	1.76

* RMS of CPs (plane precision) = $\sqrt{\frac{\sum \left(mX^{2}+mY^{2}\right)}{n}}$; ^+^ RMS of CPs (height precision) = $\sqrt{\frac{\sum mH^{2}}{n}}$.

The experimental results demonstrate that the accuracy of the novel MDCS is better than that of MACD II and that the proposed DDM calibration method is appropriate for the distortion correction of the camera with a relatively larger tilt-shift value. The system built in this study is merely a prototype system of the novel MDCS; as such, the system does not use the more accurate and expensive tilt-shift camera. Using higher accuracy TSC in the new MDCS results in further improvement of the accuracy of the photogrammetry senior product.

## 6. Conclusions

In this study, we identify the insufficiencies of traditional MDCSs and proposes a new category MDCS based on tilt-shift photography to improve ability of the MDCSs to acquire high-accuracy spatial data. We build a prototype system and use it to derive experimental expressions that illustrate how the proposed novel MDCS can be used in the field of photogrammetry and how its calibration can be conducted in the indoor control field. The characteristics of the new MDCS, via calibration and flight experiment, are listed as follows.

(1) High accuracy: experimental results show that excellent photogrammetry senior products can be obtained using the proposed MDCS.
(2) System error component: PDs and the instability of the platform need not be considered in the image processing of the novel MDCS, unlike in the traditional MDCSs.
(3) Spatial resolution unification: the composition of the proposed MDCS shows that the spatial resolution of each camera is easy to unify under the premise that the same type of camera is adopted.

The main disadvantage of the novel MDCS is the large edge distortion away from the tilt-shift direction. This study proposes a DDM approach to address this disadvantage, and additional experiment in Section 5 demonstrates that the proposed approach is more appropriate for the distortion correction of novel MDCS than the distortion coefficient method. Finally, the comparison experiments demonstrate that the proposed MDCS provide higher accuracy in acquiring spatial data (0.3 m) than MADCII (only 0.6 m) under the same conditions (spatial resolution of 0.15 m). We take the attitude that the new MDCS is a valuable addition as a sensor to the field of remote sensing and sensors.
